# The effect of two different surgical positions on pulmonary functions ın laparoscopic sleeve gastrectomies: reverse Trendelenburg vs beach chair

**DOI:** 10.1007/s00464-025-11538-2

**Published:** 2025-01-21

**Authors:** Hakan Seyit, İlke Dolğun, Erkan Bayram, Fevkiye Nur Şener, Müslüm Çiçek

**Affiliations:** 1 Department of General Surgery, Faculty of Medicine, Istınye University Medicalpark Gaziosmanpasa Hospital, Istanbul, Turkey; 2 Clinic of Anesthesiology and Reanimation, Haseki Training and Research Hospital, Istanbul, Turkey; 3 Department of Anesthesiology and Reanimation, Faculty of Medicine, Istınye University Medicalpark Gaziosmanpasa Hospital, Istanbul, Turkey; 4 Department of Internal Medicine, Faculty of Medicine, Istınye University Medicalpark Gaziosmanpasa Hospital, Istanbul, Turkey

**Keywords:** Pulmonary functions, Laparoscopic sleeve gastrectomies, Reverse Trendelenburg, Beach chair

## Abstract

**Background:**

The aim of our study is to compare the effect of the 30° reverse Trendelenburg position combined with the beach chair position on respiratory parameters in laparoscopic sleeve gastrectomy (LSG) with the 30° reverse Trendelenburg position alone.

**Material and method:**

Fifty patients with body mass index > 30 were included in the study. The patients were divided into two groups; in the control group, the standard 30° reverse Trendelenburg. In the beach chair group, the feet were positioned at 30° flexion from the hips after a 30° RTP. For both positions, blood pressures, pulses, saturations, EtCO2, respiratory rate, inspiratory pressure (Pins), positive end-expiratory pressure (PEEP), minute volume, tidal volume, peak airway pressure (Ppeak), and dynamic compliance were recorded. In addition, the general surgeon was asked about his satisfaction with the intra-abdominal operation site view and whether he was uncomfortable with the position.

**Results:**

Regardless of the group, the average age of the cases was 36.7 ± 12.1 years. There was no difference between the groups in terms of age, gender, BMI, operation time, blood pressures, heart rates, EtCO2, respiratory rate, PEEP, minute volume, tidal volume, and postoperative oxygen saturation (*p* > 0.05). Inspiratory and peak pressure were lower and dynamic compliance was higher in the beach chair position (*p* < 0.05). It was observed that the beach chair position decreased inspiratory and peak pressures and increased dynamic compliances in patients with a BMI between 35.1 and 40 (*p* < 0.05). Surgical satisfaction was high for both positions and there was no discomfort with the position.

**Conclusion:**

It was determined that the beach chair position in LSGs reduced inspiratory and peak pressures and increased dynamic compliance. These parameters were related to BMI, and the beach chair position was more positive in terms of intraoperative lung pressures and dynamic compliance, especially in patients with a BMI between 35.1 and 40.

**ClinicalTrials.gov ID: NCT06402474**.

**Supplementary Information:**

The online version contains supplementary material available at 10.1007/s00464-025-11538-2.

The laparoscopic technique has become a routine approach for many surgical procedures, especially obesity surgery, compared to traditional laparotomy techniques, as it causes less postoperative pain, shortens the hospital stay and allows patients to return to normal life more quickly [1].

In obesity, significant changes in respiratory mechanics occur compared to a normal healthy person, and this is exacerbated by general anesthesia or underlying lung diseases. These changes include decreased functional residual capacity (FRC), decreased lung and chest wall compliance, increased lung resistance, decreased oxygenation, and increased work of breathing [2].

Pneumoperitoneum and Trendelenburg position (TP) applied in laparoscopic techniques may cause a decrease in respiratory compliance, an increase in intrathoracic pressure and a decrease in functional residual volume [3, 4]. As a result, postoperative pulmonary complications such as atelectasis and ventilation/perfusion ratio incompatibility may occur in patients undergoing laparoscopic abdominal surgery [5, 6]. All these pulmonary complications become even more complicated in patients with morbid obesity, so the anesthesia care of these patients during and after laparoscopic surgery is more challenging not only for anesthesiologists but also for surgeons and pulmonologists.

Today, studies investigating various positions in laparoscopic sleeve gastrectomies (LSG) are focused on both improving the surgical field of view and reducing pulmonary complications. In the study conducted by Mulier et al. [6]; It was found that a 20° reverse Trendelenburg position (RTP) with the legs flexed at 45° hips (beach chair position) improved the surgical field of view by 770 mL, but pulmonary functions were not examined in this study. Similarly, Gao et al. [7], they found that the 30° RTP could increase static lung compliance, reduce pulmonary shunt and increase alveolo-arterial oxygen pressure and oxygenation compared to the supine position. However, the beach chair (BC) position was not studied in this study.

When we look at the literature, we have not found any studies investigating the combination of 30° RTP and BC positions in LSG. Our study was planned with the hypothesis that 30° RTP combined with the BC position could improve the patient’s cardiopulmonary functions and would not affect the surgical field of view.

The aim of our study is to investigate the effect of 30° RTP position combined with BC position on respiratory parameters in LSG.

## Material and method

The study is a prospective randomized controlled study and started after ethics committee approval İstinye University Human Research Ethics Committee decision no: 2024/03–24-80 and patients signing a written informed consent form. **ClinicalTrials.gov ID: NCT06402474**.

Criteria for inclusion in the study: Patients who could tolerate general anesthesia and pneumoperitoneum, give informed consent for surgery, were over 18 years of age, had a body mass index (BMI) > 30 kg/m^2^, and had an American Society of Anesthesiologists (ASA) physical status I–III were included. Patients who have serious underlying cardiovascular disease (e.g., congestive heart failure, conduction disorders, and ischemic heart disease); with chronic kidney disease stage 3 or greater (creatinine clearance less than 60 mL/min); have a history of cerebrovascular disease such as intracranial hypertension, cerebral infarction, cerebral hemorrhage, carotid stenosis or cerebral ischemia; have a history of chronic lung diseases such as chronic obstructive pulmonary disease, asthma, pulmonary bullae, or respiratory failure; and patients who had previous abdominal surgery, including esophagus, stomach, liver and pancreas resection, surgeries lasting longer than 90 min, patients with ASA class IV, patients with psychiatric disorders, patients who were pregnant or breastfeeding, and patients with a history of seizures were not included in the study.

### Study protocol

Randomization of the study was done by the envelope method and the patients were divided into two groups.

Group RTP: 30° reverse Trendelenburg (control) (Image 1).

Group BC: 30° reverse Trendelenburg with legs 30° hip flexion (beach chair position) (Image 2).

The intraoperative process after positioning continued with the same protocol for both groups.

### Study parameters

**Preoperative:** blood pressure, pulse, and peripheral oxygen saturation values of the patients were recorded before anesthesia induction.

**Intraoperative:** After the patients were taken to the routine operating room and standard ASA monitoring was performed, they were intubated under standard general anesthesia. Anesthesia maintenance was continued with remifentanil infusion in addition to 1–1.5 MAC sevoflurane and air mixture. Respiratory parameters were adjusted in pressure control mode, with respiratory rate and tidal volume changes, so that the end-respiratory carbon dioxide level (EtCO2) was between 30 and 35 mmHg. After the patient was draped sterile after intubation, the patient was given a randomly drawn position from the randomization envelope by an experienced personnel; Group RTP (control): standard 30° reverse Trendelenburg with feet flat (image 1); and Group BC (beach chair): after 30° reverse Trendelenburg, the feet were flexed 30° at the hips (image 2).

In both groups, while in the supine position after intubation but before the position (preop), starting from the 10th minute after the positioning and pneumoperitonium was performed (10th minute), at 10-min intervals until the end of the case, and at the 10th minute after the pneumoperitonium was terminated (70th minute); blood pressure (systolic and diastolic), pulse, peripheral oxygen saturation (SpO2), EtCO2, respiratory rate (RR), inspiratory pressure (Pins), positive end-expiratory pressure (PEEP), minute volume, tidal volume, peak airway pressure (Ppeak), and lung dynamic compliance was recorded. Pneumoperitoneum was created in all patients with carbon dioxide insufflation, keeping the maximum pressure limit up to 15 mm Hg. The general surgeon was asked about his satisfaction with the intra-abdominal operation site view (0: very dissatisfied—10: very satisfied) and whether he was uncomfortable with the position (yes/no).

The study was conducted under the same general surgeon. No patient required position change during the operation.

**Postoperative:** All patients were routinely extubated and taken to the postoperative recovery room, and blood pressure, pulse, and SpO2 were recorded at the 10th and 20th minutes postoperatively.

The primary outcome of the study is to compare the effect of 30° RTP with BC position on respiratory parameters in laparoscopic gastrectomies with 30° RTP alone.

Secondary outcomes: In addition to the effects of both positions on cardiac parameters, to investigate surgical field of view satisfaction and whether the surgeon is uncomfortable with the position.

### Statistical analysis

The power analysis of the study was based on the study of Gao et al. [7]. According to this study, arterial oxygen pressure increased in the 30° reverse Trendelenburg position compared to the supine position (262.5 ± 31.2 vs 243.1 ± 26.6). We predict that 30° RTP combined with the BC position will increase the improvement in respiratory parameters by 10%. Accordingly, in the power analysis, it was calculated that the required number of patients should be 50 in total, 25 in each group, for alpha to be 0.05 and power to be 85%. Continuous variables were shown as mean ± standard deviation values. Categorical variables are shown with numbers. The distributions of the groups were determined by Shapiro–Wilk and Kolmogorov–Smirnov tests. Independent *t* test or Mann–Whitney *U* test was used to determine the relationship between continuous variables. The relationship between categorical variables was evaluated with the chi-square test. SPSS® for Windows version 23.0 (IBM, Chicago, IL, United States) program was used in statistical analyses. Statistical significance level was accepted as *p* < 0.05.

## Results

Regardless of the group, the average age of the cases was 36.7 ± 12.1 years. The groups were similar in terms of age, gender, BMI, and operation time (Table 1).

**Table 1:** Demographic comparison of groups

	Group RTP (mean ± sd)	Group BC (mean ± sd)	p*
Age(year)	36,6 ± 13,4	36,9 ± 11,1	0,936
Sex (female/male)	16/9	17/8	0,500
BMI	43,58 ± 8,1	40,45 ± 5,8	0,124
Operation Time (minute)	69,6 ± 11,3	64,2 ± 11,3	0,099
Field of view satisfaction	8,0 ± 0,8	8,0 ± 1,2	0,791
Discomfort of position yes/no	0/25	0/25	1,000
Length of Stay (days)	2,8 ± 0,5	3,0 ± 0,5	0,102

*Student’s *t* test and Chi-Square Tests, RTP: Reverse Trendelenburg Position, BC: Beach Chair, BMI: body mass index

**Fig. 1:**
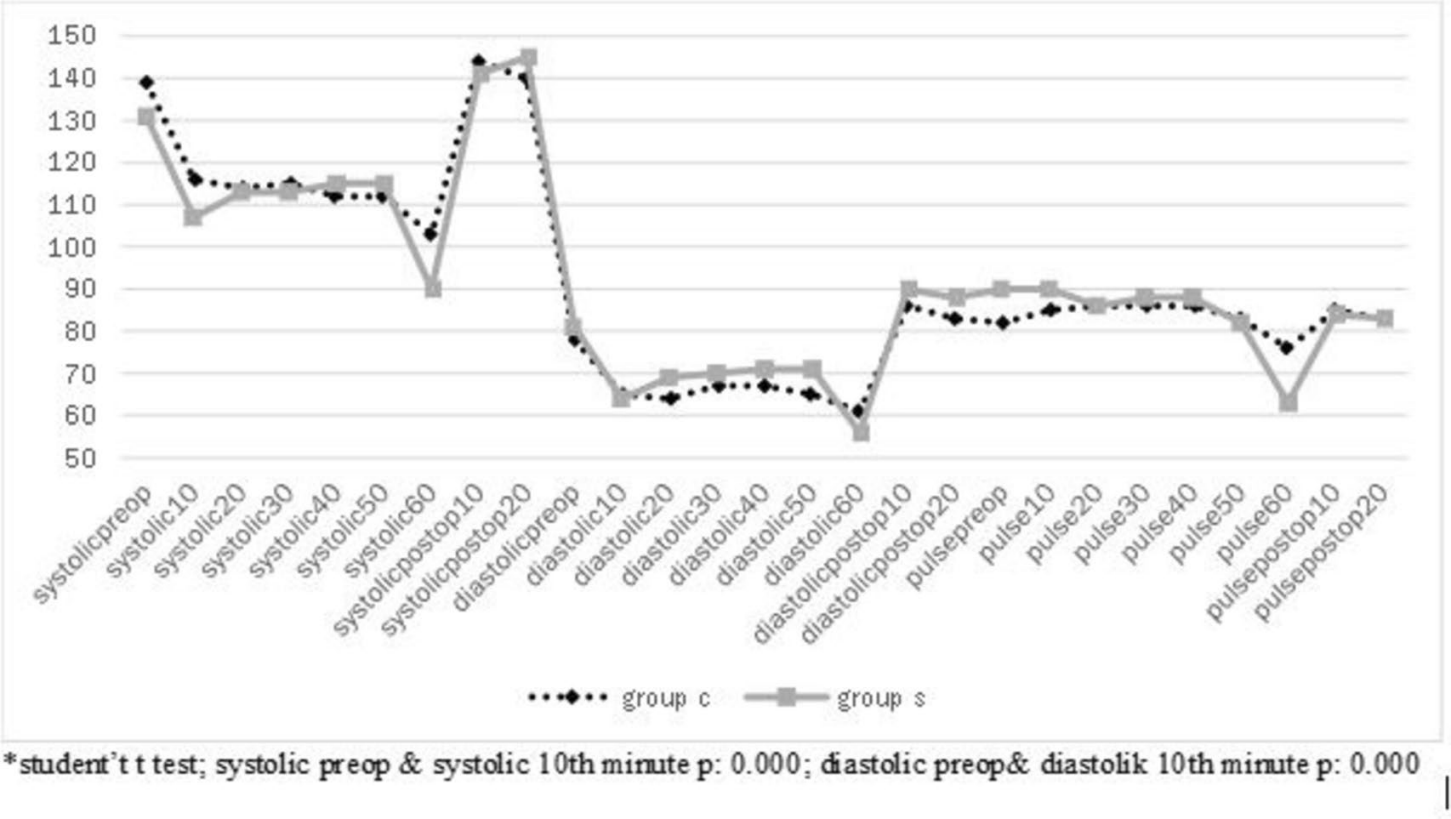
Comparison of groups’ systolic—diastolic blood pressures and heart rates over time

There was no statistically significant difference between systolic, diastolic blood pressures and heart rates between the groups (*p* > 0.05), but a significant decrease in blood pressure and a non-significant slight increase in heart rate were observed in the first 10 min after RTP in both groups (Fig. 1).

In comparing the groups in terms of respiratory parameters, there was no difference between EtCO2, respiratory rate, PEEP, minute volume, tidal volume, and postoperative oxygen saturation (*p* > 0.05, Table 2). However, when we looked at the changes over time, it was determined that EtCO2 decreased at the 10th minute in both groups compared to preoperative values. Tidal volume increased significantly at the 10th minute compared to preoperative values only in the BC position. Additionally, postoperative oxygen saturations in both groups were lower than preoperative values, but after the abdomen was deflated (70th minute), oxygen saturation increased significantly in both groups (*p* < 0.05, Table 2).

**Table 2:** Intergroup comparison of preoperative respiratory changes

	Group RTP (mean ± sd)	p^2^ (preop & 10. Minute)	Group BC (mean ± sd)	p^1^	p^2^ (preop & 10. Minute)
EtCO2-preop	36,6 ± 8,7	0,000*	33,5 ± 7,6	0,190	0,000*
EtCO2-10	32,9 ± 4,5		32,2 ± 5,6	0,642	
EtCO2-20	33,3 ± 3,6		35,8 ± 5,6	0,065	
EtCO2-30	33,3 ± 3,9		35,9 ± 4,3	0,090	
EtCO2-40	33,6 ± 3,3		35,6 ± 4,8	0,059	
EtCO2-50	33,7 ± 3,4		34,8 ± 5,2	0,358	
EtCO2-60	33,6 ± 3,6		36,0 ± 5,1	0,383	
EtCO2-70	33,8 3,8		34,1 4,6	0,864	
RR-preop	12,8 ± 1,6	0,325	12,0 ± 2,7	0,155	0,958
RR-10	13,0 ± 1,9		12,0 ± 1,2	0,248	
RR-20	13,1 ± 1,9		12,8 ± 1,9	0,558	
RR-30	13,3 ± 2,1		12,8 ± 1,8	0,438	
RR-40	13,3 ± 2,3		12,7 ± 1,5	0,295	
RR-50	13,3 ± 2,3		12,5 ± 1,1	0,141	
RR-60	13,6 ± 4,4		12,6 ± 1,7	0,257	
RR-70	13,9 2,9		12,0 1,0	0,069	
PEEP-preop	5,0 ± 0,7	0,999	5,0 ± 0,5	0,990	0,982
PEEP-10	5,0 ± 0,7		5,0 ± 0,9	0,873	
PEEP-20	5,1 ± 0,6		5,0 ± 0,6	0,672	
PEEP-30	5,5 ± 1,2		5,1 ± 0,7	0,226	
PEEP-40	5,3 ± 1,2		5,1 ± 0,9	0,460	
PEEP-50	5,4 ± 1,1		5,0 ± 0,8	0,181	
PEEP-60	5,4 ± 1,9		5,4 ± 1,2	0,692	
PEEP-70	5,4 1,5		4,8 0,3	0,296	
Minute volume.-preop	8,1 ± 1,2	0,963	8,6 ± 1,1	0,427	0,363
Minute volume- 10	8,1 ± 1,9		7,8 ± 1,6	0,543	
Minute volume-20	8,4 ± 1,6		8,0 ± 1,5	0,349	
Minute volume-30	8,3 ± 1,6		8,5 ± 1,6	0,792	
Minute volume-40	8,5 ± 1,7		8,5 ± 1,4	0,958	
Minute volume-50	8,6 ± 1,4		8,8 ± 1,4	0,575	
Minute volume-60	8,8 ± 1,2		8,6 ± 1,3	0,412	
Minute volüme-70	8,6 1,8		8,1 1,0	0,437	
Tidal volume-preop	611,1 ± 88,3	0,125	627,2 ± 113,0	0,545	0,010*
Tidal volume-10	618,0 ± 114,2		676,3 ± 142,1	0,117	
Tidal volume-20	638,4 ± 83,1		630,4 ± 142,9	0,811	
Tidal volume-30	624,7 ± 99,3		669,7 ± 122,3	0,160	
Tidal volume-40	644,4 ± 111,6		672,2 ± 117,1	0,396	
Tidal volume-50	647,6 ± 105,6		698,4 ± 115,8	0,112	
Tidal volume-60	649,6 ± 112,4		692,8 ± 114,6	0,298	
Tidal volüme-70	623,1 126,0		671,5 76,9	0,308	

		**p**^**3**^ **Preop&70, Preop&postop10 and postop10&postop20**			**p**^**3**^ **Preop&70, Preop&postop10 and postop10&postop20**
SpO2 preop	97,8 ± 1,8	0,000*	97,8 ± 1,6	0,875	0,000*
SpO2-70	98,8 1,4		99,4 1,0	0,299	
SpO2 postop10	96,64 ± 2,2		94,6 ± 2,5	0,548	
SpO2 postop20	97,3 ± 1,7		96,2 ± 1,7	0,931	

*Student’s *t* test. The mean difference is significant at the 0.05 level. p^1^: group RTP & group BC; p^2^: preop & 10. Minute; p^3^: Preop&70, Preop&postop10, and postop10&postop20 for SpO2; RTP: Reverse Trendelenburg Position; BC: Beach Chair; EtCO2: end-tidal carbon dioxide, RR: respiratory rate, PEEP: positive end-expiratory pressure, SpO2postop: postoperative oxygen saturation,

In comparing pressures from respiratory parameters, a statistically significant difference was detected between the groups in terms of inspiratory pressure (Pins), peak pressure (PPeak) and dynamic compliance throughout the operation period. According to our findings, pressures such as Pins and Ppeak were lower in the BC position, and dynamic compliance was significantly higher (*p* < 0.05, Table 3). Similarly, when we evaluated these parameters according to time within the group, the 10th minute after positioning was the time interval in which Pins and Ppeak decreased the most and dynamic compliance increased the most, according to preoperative values in both groups (*p* < 0.05, Table 3). There was no difference in these parameters between the 70th minute when pneumoperitoneum was terminated and the previous 60th minute.

**Table 3:** Intergroup comparison of preoperative pulmonary pressure changes

	Group RTP (mean ± sd)	Δ (difference from Pins-preop to each timepoint)	p^2^ (preop & 10. Minute)	Group BC (mean ± sd)	Δ (difference from Pins-preop to each timepoint)	p^1^ (Δ)	p^2^ (preop & 10. Minute)
Pins-preop	27,5 ± 3,7		0,000*	25,5 ± 4,3		0,091	0,000*
Pins-10	25,5 ± 3,4	2 ± 0,3		21,6 ± 4,1	3,9 ± 0,2	0,001*	
Pins-20	25,9 ± 3,0	1,6 ± 0,7		22,2 ± 3,3	3,3 ± 1,0	0,000*	
Pins-30	25,9 ± 3,0	1,6 ± 0,7		23,2 ± 3,6	2,3 ± 0,7	0,006*	
Pins-40	25,9 ± 3,2	1,6 ± 0,5		23,6 ± 3,6	1,9 ± 0,7	0,043*	
Pins-50	25,7 ± 3,1	1,8 ± 0,6		23,5 ± 3,6	2,0 ± 0,7	0,126	
Pins-60	25,7 ± 3,7	1,8 ± 0,0		22,7 ± 2,8	2,8 ± 1,5	0,013*	
Pins-70	25,7 ± 3,3	1,8 ± 0,4		21,7 ± 1,9	3,8 ± 2,4	0,003*	
Ppeak-preop	26,8 ± 3,1		0,027*	24,2 ± 3,2		0,142	0,000*
Ppeak-10	25,4 ± 3,5	1,4 ± 0,4		22,0 ± 3,9	2,2 ± 0,7	0,003*	
Ppeak-20	25,7 ± 3,0	1,1 ± 0,1		22,5 ± 3,5	1,7 ± 0,3	0,001*	
Ppeak-30	25,7 ± 2,9	1,1 ± 0,2		23,2 ± 3,3	1,0 ± 0,1	0,118*	
Ppeak-40	25,7 ± 3,3	1,1 ± 0,2		23,7 ± 3,4	0,5 ± 0,2	0,039*	
Ppeak-50	25,5 ± 3,1	1,3 ± 0,0		23,8 ± 3,4	0,4 ± 0,2	0,022*	
Ppeak-60	25,5 ± 3,0	1,3 ± 0,1		23,3 ± 3,6	0,9 ± 0,4	0,036*	
Ppeak-70	25,1 ± 3,7	0,3 ± 0,6		22,7 ± 2,4	1,5 ± 0,8	0,001*	
Dyncomp-preop	28,0 ± 5,5		0,000*	29,5 ± 7,1		0,514	0,000*
Dyncomp-10	31,3 ± 6,2	3,3 ± 0,7		39,5 ± 7,9	10,0 ± 0,8	0,000*	
Dyncomp-20	31,7 ± 5,8	3,7 ± 0,3		37,4 ± 7,0	7,9 ± 0,1	0,003*	
Dyncomp-30	31,8 ± 5,6	3,8 ± 0,1		37,6 ± 7,8	8,1 ± 0,7	0,004*	
Dyncomp-40	31,5 ± 5,7	3,5 ± 0,2		38,6 ± 7,4	9,1 ± 0,3	0,001*	
Dyncomp-50	32,3 ± 5,8	4,3 ± 0,3		41,3 ± 9,9	11,8 ± 2,8	0,000*	
Dyncomp-60	32,0 ± 5,9	4,0 ± 0,4		40,3 ± 8,1	10,8 ± 1,0	0,000*	
Dyncomp-70	32,0 ± 6,2	4,0 ± 0,7		41,4 ± 6,6	11,9 ± 0,5	0,000*	

*Student’s *t* test. The mean difference is significant at the 0.05 level. p^1^: group RTP & group BC for **Δ**; p^2^: preop & 10. Minute; RTP: Reverse Trendelenburg Position; BC: Beach Chair; Pins: inspiratory pressure, Ppeak: airway peak pressure, Dyncomp: dynamic compliance (only statistically significant parameters are shown)

When the situations related to this statistical significance in pressures were investigated, it was found that it was only related to BMI. We grouped BMI to understand how BMI affects these pressures. When we grouped it as 30–35, 35.1–40, and 40.1 and above and looked at its effect on intergroup pressures, it was seen that the BC position decreased the inspiratory and peak pressures and increased the dynamic complication in patients with BMI: 35.1—40 (*p* < 0.05, Table 4). However, when the effect of BMI differences within the groups on pressures was examined, no significant difference was detected.

**Table 4:** Comparison of intergroup pressure changes according to BMI

Group of BMI		Group RTP (n:9) (mean ± sd)	Group BC (n:8) (mean ± sd)	p*
35,1–40	Pins-preop	27,8 ± 3,5	25,4 ± 4,4	0,102
	Pins-10	24,8 ± 3,3	20,8 ± 4,1	0,049*
	Pins-20	25,4 ± 2,9	21,0 ± 2,9	0,007*
	Pins-30	25,3 ± 2,8	21,2 ± 2,6	0,008*
	Pins-40	25,5 ± 2,9	22,1 ± 2,5	0,022*
	Pins-50	25,3 ± 2,9	22,2 ± 2,5	0,028*
	Pins-60	25,4 ± 3,0	22,3 ± 2,8	0,024*
	Ppeak-preop	26,7 ± 3,1	24,6 ± 3,2	0,157
	Ppeak-10	24,7 ± 3,2	21,1 ± 3,7	0,049*
	Ppeak-20	25,3 ± 2,7	21,0 ± 2,7	0,006*
	Ppeak-30	25,2 ± 2,7	21,5 ± 2,3	0,009*
	Ppeak-40	25,2 ± 3,2	22,1 ± 2,5	0,043*
	Ppeak-50	25,2 ± 2,8	22,2 ± 2,4	0,037*
	Ppeak-60	25,0 ± 3,0	22,1 ± 2,3	0,026*
	Dyncomp-preop	28,0 ± 4,9	29,5 ± 6,8	0,453
	Dyncomp-10	31,4 ± 7,0	44,7 ± 6,2	0,001*
	Dyncomp-20	32,8 ± 7,4	43,2 ± 6,8	0,009*
	Dyncomp-30	32,4 ± 7,0	42,6 ± 7,9	0,014*
	Dyncomp-40	32,8 ± 6,6	45,0 ± 7,6	0,003*
	Dyncomp-50	32,1 ± 6,4	48,0 ± 11,3	0,001*
	Dyncomp-60	32,5 ± 7,1	44,6 ± 6,9	0,010*

		Group RTP (n:13)	Group BC (n:11)	
≥ 40,1	Pins-preop	28,0 ± 3,3	26,1 ± 4,1	0,122
	Pins-10	26,5 ± 3,6	22,4 ± 4,2	0,019*
	Pins-20	26,8 ± 3,1	23,2 ± 3,4	0,015*
	Pins-30	27,0 ± 3,0	24,6 ± 3,9	0,112
	Pins-40	26,8 ± 3,3	25,0 ± 4,2	0,244
	Pins-50	26,6 ± 3,3	24,8 ± 4,0	0,245
	Pins-60	24,4 ± 3,7	23,7 ± 3,5	0,120
	Ppeak-preop	28,1 ± 3,7	26,2 ± 3,7	0,213
	Ppeak-10	26,3 ± 3,8	23,1 ± 4,2	0,072
	Ppeak-20	26,6 ± 3,0	24,0 ± 3,5	0,066
	Ppeak-30	26,6 ± 2,9	24,6 ± 3,4	0,126
	Ppeak-40	26,7 ± 3,3	25,2 ± 3,5	0,302
	Ppeak-50	26,4 ± 3,2	25,6 ± 3,5	0,561
	Ppeak-60	25,8 ± 3,0	25,0 ± 3,7	0,185
	Dyncomp-preop	27,9 ± 4,1	30,1 ± 5,9	0,153
	Dyncomp-10	30,7 ± 6,1	36,4 ± 7,2	0,055
	Dyncomp-20	30,6 ± 4,8	34,1 ± 5,6	0,110
	Dyncomp-30	30,7 ± 4,7	33,5 ± 5,7	0,208
	Dyncomp-40	29,6 ± 4,4	33,6 ± 5,1	0,056
	Dyncomp-50	31,0 ± 3,9	36,0 ± 5,4	0,015*
	Dyncomp-60	30,9 ± 4,1	34,0 ± 5,6	0,158

*Student’s* t* test. The mean difference is significant at the 0.05 level

There was no significant difference in surgeon satisfaction with field of view and discomfort with the position. Surgical satisfaction was high for both positions (Table 1).

## Dıscussıon

In this study, the effect of RTP and BC position on intraoperative pulmonary functions in LGS, which is encountered in increasing numbers in the treatment of obesity. While there was no difference between the two positions in terms of hemodynamic (blood pressure and heart rate) and respiratory (EtCO2, PEEP, minute volume, tidal volume, SpO2) parameters, inspiratory and peak pressures were significantly lower in the BC position and dynamic compliance was higher in the BC position. It was observed that these three parameters were only related to BMI and that the BC position was more ameliorative in terms of intraoperative pressures and dynamic compliance, especially in patients with a BMI between 35.1 and 40. In addition, the surgeon’s field of view satisfaction was the same in both positions and there was no discomfort in either position.

Laparoscopic bariatric procedures in obese patients; it requires creating pneumoperitoneum and maintaining patients in RTP, which is known to cause changes in cardiac output and peripheral tissue perfusion. RTP under general anesthesia may cause hypotension due to decreased preload caused by venous pooling in the lower extremities and pelvis [8]. As intra-abdominal pressure increases, systemic vascular resistance increases as a result of mechanical compression of the abdominal aorta, release of neurohumoral hormones, such as vasopressin, and activation of the renin–angiotensin–aldosterone axis. Narrowing of the inferior vena cava reduces preload, which may result in decreased cardiac output and therefore decreased arterial pressure. In our study, a significant decrease in blood pressure and a slight increase in heart rate were observed in both groups after positioning and creating pneumoperitoneum. Our findings were consistent with the literature [9–11], but there was no difference between blood pressure and heart rates in comparisons in terms of two different positions.

Although obesity places greater demands on the respiratory system to maintain normal PaCO2, most patients with severe obesity are eucapnic [12]. In simple obesity, tidal volume is normal both at rest and during maximum exercise. In our study, the patients were eucapnic in the preoperative period. During laparoscopic operations, pneumoperitoneum is created by CO2 insufflation. During pneumoperitoneum, factors such as intraperitoneal CO2 absorption, increased intra-abdominal pressure, patient position, artificial ventilation, as well as anesthesia can affect respiratory mechanics and blood gases [13]. In this study, there was no significant difference between two different positions in terms of respiratory rate, PEEP, minute volume, EtCO2, and tidal volume, among the parameters that can be adjusted to maintain eucapnia under anesthesia and pneumoperitoneum and to provide adequate tidal volume according to ideal body weight. Although many obesity studies in the literature are aimed at finding ideal PEEP [14–16], in our study, we aimed to investigate the effect of BC position alone on respiratory mechanics by keeping PEEP as constant as possible and observed that BC position has no effect on the parameters mentioned.

Obese patients are more likely to develop intraoperative atelectasis because they exhibit a decrease in functional residual capacity (FRC) during general anesthesia [17]. Atelectasis is one of the main causes of postoperative hypoxemia (PH) and may predispose to adverse postoperative outcomes, such as respiratory failure, pneumonia, and mortality [18]. In our study, in line with the literature [12, 19], postoperative oxygen saturations were significantly lower than preoperative values ​​in both groups, but there was no difference between the two groups. They approached their initial values ​​with nasal oxygen support. As we mentioned before, we attributed this situation to both general anesthesia, the inability to breathe strongly due to postoperative pain, and atelectasis caused by the increase in intra-abdominal and intrathoracic pressure due to the pneumoperitoneum created by CO2 insufflation [12]. Our data supporting this finding was that when pneumoperitoneum was terminated, oxygen saturations increased significantly in both groups, being more in the BC group. Despite relative intraoperative hypoxemia, no serious clinical respiratory complications were reported in the postoperative period.

It is generally accepted that respiratory system compliance is low in obesity due to the effect of obesity on the chest wall. Lung compliance decreases by approximately 25% in simple obesity. Part of the decrease is probably due to deterioration of lung elasticity due to increased pulmonary blood volume and part of it is due to the decrease in FRC. In addition, these patients are characterized by an increase in respiratory resistance. The most important reason for increased lung and respiratory resistance is the decrease in lung volume [12]. Based on all these data, it is an undeniable fact that the apparent irregularity in the FRC, elastic (decrease in lung and chest wall compliance), and resistive (increase in lung resistance) components of the respiratory system may be responsible for the significant respiratory dysfunction and arterial hypoxemia that occur in the postoperative period. Therefore, methods to improve respiratory mechanics in such patients are needed. In our present study, we found that the BC position reduced inspiratory and peak pressures significantly more than RTP alone and improved dynamic compliance by 33.9% versus 11.8%. Although there is no study in the literature similar to our study comparing two positions, we come across data to support our findings in individual position studies. In the study conducted by Gao et al. [7], they suggested that 30 degrees reverse Trendelenburg can increase static lung compliance, reduce pulmonary shunt, and increase arterial oxygen pressure and oxygenation compared to the supine position. Valenza et al. [14] claimed that lung volumes almost doubled in the beach chair position, airway pressures decreased, oxygenation and respiratory mechanics improved, and when applied with PEEP, they managed to eliminate the harmful effects of pneumoperitoneum. Another finding that attracted our attention in our study was that the effects of pneumoperitoneum continued to be seen in the measurements 10 min after the intra-abdominal pressure was relieved. We believe that this is a subject open to research.

It is known that lung resistance and changes in respiratory mechanics are related to BMI [12, 20]. As BMI increases, lung resistance also increases and a decrease in the compliance of the respiratory system is observed [20]. In our study, similar to the literature, we found that as BMI increased, inspiratory and peak pressures, in other words resistances, increased and dynamic compliance decreased, regardless of the group. The finding that caught our attention was that the BC position improved respiratory mechanics more in patients with BMI 35.1–40 than in other BMI groups. In light of this finding, we believe that the BC position may be beneficial in certain patient groups. However, this finding should be supported by other studies.

It is important for the surgeon to have sufficient intra-abdominal working area during laparoscopic surgery in obese patients. Most surgeons use an inflation pressure of 15 mmHg. Müljer et al. [6] calculated the minimum intra-abdominal volume required for laparoscopic upper abdominal surgery as 3 L and stated that the field of view is restricted in smaller volumes. In the same study, they found that there was sufficient working space in 50% of patients in the reverse Trendelenburg position and in 80% of patients in the beach chair position, that the BC position helped improve the surgical working area in cases where the body position was not sufficient, and that it was the most effective method in improving the working area by 770 mL after complete muscle relaxation. Additionally, they stated that the beach chair position did not disturb the surgeon, who remained between open and raised legs while performing laparoscopic upper abdominal surgery. Although surgical field of view measurement was not performed in our study, the surgeon’s field of view satisfaction was 8 or above in both positions and they stated that they were not disturbed by the position.

There are some limitations in our study. First of all, our sample size is not sufficient to generalize the application of BC position. Since our study is the first comparison study in the literature, more randomized controlled studies with a larger number of cases are needed. Especially, a larger sample size and more advanced parameters (such as blood gas analysis) are needed to make a general conclusion about why respiratory parameters did not change in the 30–35 BMI and 40.1 and above BMI groups. Another limitation of ours is that we were not able to measure lung volumes and blood gases due to financial concerns. Another limitation is that we did not monitor respiratory mechanics in the early postoperative period, only the first 20 min of the postoperative period. There is a need for studies that consider the intraoperative and postoperative pulmonary effects of the BC position together. Since it was not among our primary objectives, 30-day complications were not investigated. However, future studies may be in this direction. Additionally, the study was conducted by the same surgeon. Therefore, it cannot be concluded from this article that other surgeons may also use the split-leg position.

## Conclusion

In this study, the effect of RTP and BC positions in LSGs on intraoperative pulmonary functions was investigated. There was no difference between the two positions in terms of hemodynamic and respiratory parameters. It was observed that inspiratory and peak pressures (resistance) were lower and dynamic compliance was higher in the BC position. It was observed that these parameters were related to BMI and that the BC position was more ameliorative in terms of intraoperative pressures and dynamic compliance, especially in patients with a BMI between 35.1 and 40. In light of our findings, we believe that the BC position may be preferred in terms of lung-protective ventilation strategies.

## Supplementary Information

Below is the link to the electronic supplementary material.

Supplementary file1 (DOC 52 KB)

Image 1: Group RTP (control): standard 30° reverse Trendelenburg with feet flat
Supplementary file2 (JPG 3199 KB)

Image 2: Group BC (beach chair): After 30° reverse Trendelenburg, the feet were flexed 30° at the hips
Supplementary file3 (JPG 2562 KB)
