# Supplementary material for: The effect of two different surgical positions on pulmonary functions ın laparoscopic sleeve gastrectomies: reverse Trendelenburg vs beach chair
Source: Surg Endosc. 2025 Jan 21;39(3):1829–38. doi: 10.1007/s00464-025-11538-2 (PMC11870926; doi:10.1007/s00464-025-11538-2)
Supplement: Supplementary file 1 — Supplementary file1 (DOC 52 KB) [file 464_2025_11538_MOESM1_ESM.doc]

**
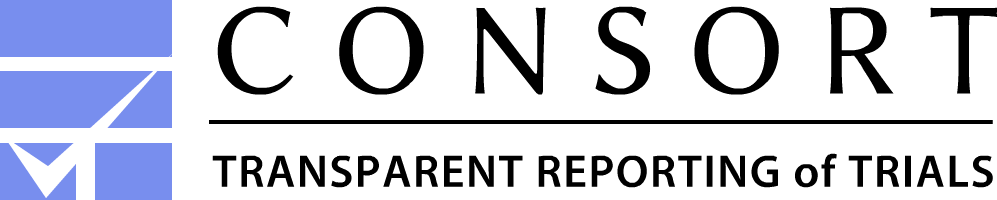
**

**CONSORT 2010 Flow Diagram**

**Allocation**

**Analysis**

**Follow-Up**

**Enrollment**

Assessed for eligibility (n= 50)

Excluded (n= 0)

Analysed (n= 25 )

n=25

Allocated to intervention (n=25)

♦ Group RTP: 30° reverse trendelenburg (control)

n=25

Allocated to intervention (n= 25)

Group BC: 30° reverse trandelenburg with legs 30° hip flexion (beach chair position)

Analysed (n= 25 )

Randomized (n= 50)
