# Supplementary figures and images for: The effect of two different surgical positions on pulmonary functions ın laparoscopic sleeve gastrectomies: reverse Trendelenburg vs beach chair
Source: Surg Endosc. 2025 Jan 21;39(3):1829–38. doi: 10.1007/s00464-025-11538-2 (PMC11870926; doi:10.1007/s00464-025-11538-2)

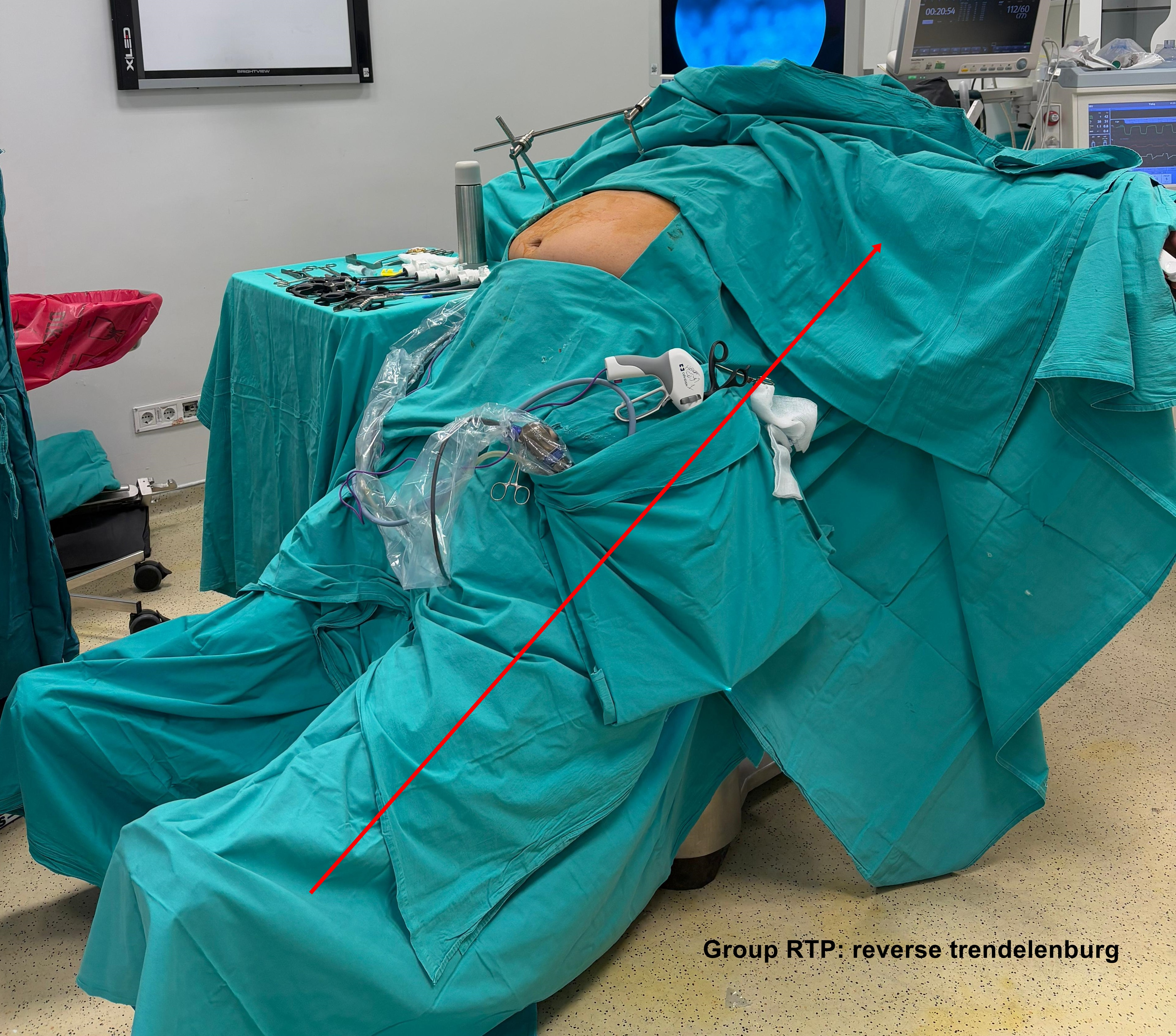

Supplement: Supplementary file 2 — Image 1: Group RTP (control): standard 30° reverse Trendelenburg with feet flat Supplementary file2 (JPG 3199 KB) [file 464_2025_11538_MOESM2_ESM.jpg]

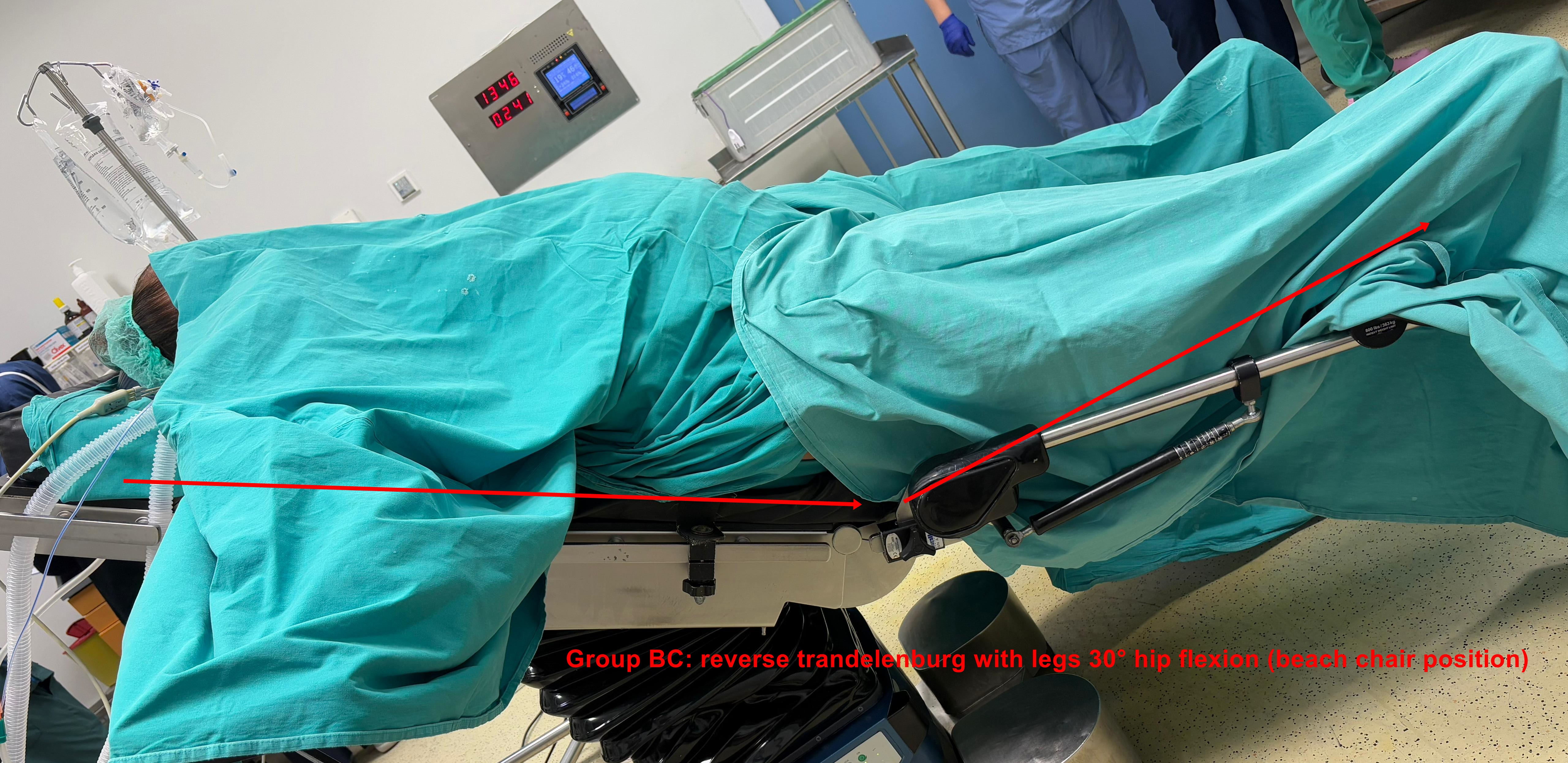

Supplement: Supplementary file 3 — Image 2: Group BC (beach chair): After 30° reverse Trendelenburg, the feet were flexed 30° at the hips Supplementary file3 (JPG 2562 KB) [file 464_2025_11538_MOESM3_ESM.jpg]
